# Antibiotic prophylaxis for percutaneous renal biopsy: study protocol for a prospective randomized trial

**DOI:** 10.1186/s13063-022-06618-w

**Published:** 2022-08-11

**Authors:** Kensei Yahata, Akihiro Yoshimoto, Chiharu Kinoshita, Koichi Seta, Tatsuo Tsukamoto, Motoko Yanagita, Hiroaki Hata, Naoki Sakane

**Affiliations:** 1 Department of Nephrology, Osaka Red Cross Hospital, Osaka, Japan; 2 Department of Nephrology, Kobe City Medical Center General Hospital, Kobe, Japan; 3 Department of Nephrology, Kyoto Min-Iren Chuo Hospital, Kyoto, Japan; 4 Department of Nephrology, National Hospital Organization Kyoto Medical Center, Kyoto, Japan; 5 Center of Nephrology and Urology, Division of Nephrology and Dialysis, Kitano Hospital, The Tazuke Kofukai Medical Research Institute, Osaka, Japan; 6 Department of Nephrology, Graduate School of Medicine, Kyoto University, Kyoto, Japan; 7 Department of Surgery, National Hospital Organization Kyoto Medical Center, Kyoto, Japan; 8 Division of Translational Research, National Hospital Organization Kyoto Medical Center, Kyoto, Japan

**Keywords:** Antibiotic prophylaxis, Percutaneous renal biopsy, Randomized controlled trial

## Abstract

**Background:**

The major complication of renal biopsy is bleeding. Infection is an extremely rare complication of percutaneous renal biopsy, providing sterile techniques are used and bowel perforation does not occur. However, the questionnaire included in the Kidney Biopsy Guidebook 2020 in Japan reported that antibiotic prophylaxis was administered to patients undergoing percutaneous renal biopsy at 61% of 170 adult institutions and 57% of 54 pediatric institutions. The objective of this study is to show the non-inferiority of not administering antibiotic prophylaxis for percutaneous renal biopsy.

**Methods:**

Patients aged ≥15 years who are scheduled to undergo percutaneous renal biopsy are eligible for inclusion in the study. Three hundred and sixty-four patients will be recruited at 6 hospitals. The patients will be randomly assigned at a 1:1 ratio to receive either a single dose of intravenous cefazolin (1 g) or no antibiotic prophylaxis. The primary outcome is the number of patients that exhibit positive urine cultures (>10^5^ colony-forming units/ml) 3 or 4 days after the renal biopsy, or at which point the patients are diagnosed with pyelonephritis until 3 or 4 days after the renal biopsy. The secondary outcomes are the number of patients who are diagnosed with pyelonephritis within 30 days after the renal biopsy, the number of patients who are diagnosed with puncture site infections within 30 days after the renal biopsy, the number of patients who are diagnosed with an infection other than pyelonephritis or a puncture site infection within 30 days after the renal biopsy, and the number of patients who experience cefazolin-induced side effects.

**Discussion:**

This randomized controlled trial aims to show the non-inferiority of not administering antibiotic prophylaxis for percutaneous renal biopsy. If this study shows that antibiotic prophylaxis is not needed, it would help to ensure patient safety and prevent the development of antibiotic-resistant bacteria.

**Trial registration:**

UMIN Clinical Trials Registry (UMIN-CTR) UMIN000042378. Registered on 7 Nov 2020.

**Supplementary Information:**

The online version contains supplementary material available at 10.1186/s13063-022-06618-w.

## Administrative information

Note: the numbers in curly brackets in this protocol refer to SPIRIT checklist item numbers. The order of the items has been modified to group similar items (see http://www.equator-network.org/reporting-guidelines/spirit-2013-statement-defining-standard-protocol-items-for-clinical-trials/).

**Table:** 

Title {1}	Antibiotic prophylaxis for percutaneous renal biopsy: study protocol for a prospective randomized trial
Trial registration {2a and 2b}.	UMIN000042378 [UMIN Clinical Trials Registry (UMIN-CTR)] http://www.umin.ac.jp/ctr [Registered on 7^th^ November 2020]
Protocol version {3}	Version 3 of 15^th^ November 2021
Funding {4}	This study was supported by donations from Osaka Red Cross Hospital and National Hospital Organization Kyoto Medical Center. We did not receive any funds that were specifically targeted at this study.
Author details {5a}	Kensei Yahata: Department of Nephrology, Osaka Red Cross Hospital, Osaka, Japan Akihiro Yoshimoto: Department of Nephrology, Kobe City Medical Center General Hospital, Kobe, Japan Chiharu Kinoshita: Department of Nephrology, Kyoto Min-Iren Chuo Hospital, Kyoto, Japan Koichi Seta: Department of Nephrology, National Hospital Organization Kyoto Medical Center, Kyoto, Japan Tatsuo Tsukamoto: Center of Nephrology and Urology, Division of Nephrology and Dialysis, Kitano Hospital, The Tazuke Kofukai Medical Research Institute, Osaka, Japan Motoko Yanagita: Department of Nephrology, Graduate School of Medicine, Kyoto University, Kyoto, Japan Hiroaki Hata: Department of Surgery, National Hospital Organization Kyoto Medical Center, Kyoto, Japan Naoki Sakane: Division of Translational Research, National Hospital Organization Kyoto Medical Center, Kyoto, Japan
Name and contact information for the trial sponsor {5b}	Osaka Red Cross Hospital: soumudaiichi@osaka-med.jrc.or.jp National Hospital Organization Kyoto Medical Center: 404-syomu2@mail.hosp.go.jp
Role of sponsor {5c}	The sponsors played no role in the design of the study and will not play any role in the collection, management, analysis, or interpretation of the data. They do not have ultimate authority over any of these activities.

## Introduction

### Background and rationale {6a}

Renal biopsy is the most important technique in the field of nephrology and is used to pathologically diagnose renal disease, estimate prognoses and the effects of treatment, and determine the optimal treatment strategy. The major complication of renal biopsy is bleeding. On the other hand, infection is an extremely rare complication of percutaneous renal biopsy, providing a sterile technique is used and bowel perforation does not occur [1]. However, the questionnaire included in the Kidney Biopsy Guidebook 2020 in Japan showed that antibiotic prophylaxis was administered to patients undergoing percutaneous renal biopsy at 61% of 170 adult institutions and 57% of 54 pediatric institutions [2].

The guidelines developed jointly by the American Society of Health-System Pharmacists (ASHP), the Infectious Diseases Society of America (IDSA), the Surgical Infection Society (SIS), and the Society for Healthcare Epidemiology of America (SHEA) included policies relating to the administration of antibiotic prophylaxis in various types of surgery [3]. According to these guidelines, the use of antibiotic prophylaxis is not recommended for some types of clean and low-infectious-risk surgery. In addition, it is stated that postoperative antimicrobial treatment is not necessary after most procedures, and the duration of antimicrobial prophylaxis should be less than 24 h for most procedures. The 2017 Centers of Disease Control and Prevention guidelines recommend that antibiotic prophylaxis should ensure that bactericidal concentrations of the antibiotic are achieved in blood and tissue before a skin incision is made [4]. Moreover, it is stated that antibiotic prophylaxis should not be administered after an incision has been closed in clean surgery. The World Health Organization guidelines state that antibiotic prophylaxis should be administered within 120 minutes before skin incisions, and they oppose the continuing administration of antibiotic prophylaxis after surgery [5].

The questionnaire included in the Kidney Biopsy Guidebook 2020 in Japan did not examine the type or administration route of antibiotic prophylaxis, e.g., whether the drug was orally administered for several days or intravenously injected before the renal biopsy. In addition, no previous studies have examined the efficacy of antibiotic prophylaxis for percutaneous renal biopsies, regardless of the type or route of the prophylaxis.

### Objectives {7}

The objective of this study was to show the non-inferiority of not administering antibiotic prophylaxis for percutaneous renal biopsy. If this study shows that antibiotic prophylaxis is not needed for percutaneous renal biopsy, it would help to ensure patient safety and prevent the development of antibiotic-resistant bacteria.

### Trial design {8}

This is a multicenter randomized controlled trial. The participants will be randomly assigned at a 1:1 ratio to receive either a single dose of intravenous cefazolin (1 g) or no antibiotic prophylaxis (cefazolin versus no prophylaxis group).

## Methods: participants, interventions, and outcomes

### Study setting {9}

This study will be performed at Osaka Red Cross Hospital, Kobe City Medical Center General Hospital, Kyoto Min-Iren Chuo Hospital, National Hospital Organization Kyoto Medical Center, Kitano Hospital, and Kyoto University Hospital. Patients will be considered for inclusion if they meet the criteria outlined below.

### Eligibility criteria {10}

#### Inclusion criteria

- Patients who are aged ≥15 years who are scheduled to undergo percutaneous renal biopsy

#### Exclusion criteria

- Patients who present with a white blood cell count of >10/high-power field (HPF) on two occasions during the most recent urinalysis (including once within 3 days of the procedure)
- Patients who are allergic to cefazolin
- Patients who receive antibiotics in the 7 days before the renal biopsy
- Patients who undergo urethral catheter insertion in the 7 days before the renal biopsy
- Patients infected with human immunodeficiency virus
- Pregnant patients

### Who will take informed consent? {26a}

The doctors who perform the percutaneous renal biopsies will identify eligible participants based upon the inclusion and exclusion criteria. Detailed written and oral information on the study will be provided to the patients, and the doctors will obtain written informed consent.

#### Additional consent provisions for collection and use of participant data and biological specimens {26b}

On the consent form, participants will be asked if they agree to the use of their data should they choose to withdraw from the study. The participants will also be asked for permission for the study team to share relevant data with people from the universities taking part in the study or with regulatory authorities, where relevant. This study does not involve collecting biological specimens for storage.

## Interventions

### Explanation for the choice of comparators {6b}

Antibiotic prophylaxis is administered at the majority of institutions in Japan. Therefore, we decided to use a cefazolin group as a comparator.

### Intervention description {11a}

The objective of this study is to show the non-inferiority of not administering antibiotic prophylaxis for percutaneous renal biopsies. Therefore, we decided to use a no prophylaxis group as the intervention group.

### Criteria for discontinuing or modifying allocated interventions {11b}

Patients can leave the study at any time for any reason if they wish to do so without any consequences. Any patient data that have been collected up to that moment will be included in the analysis. When a side effect occurs during cefazolin administration, the administration of the drug will be discontinued. Patients that become ill will be asked to contact the primary investigator.

### Strategies to improve adherence to interventions {11c}

Patients will remain in close contact with the doctors, who will monitor their progression during study visits. If a patient does not come to the hospital as scheduled, we will contact them about their condition via telephone or a sealed letter.

### Relevant concomitant care permitted or prohibited during the trial {11d}

Any care is permitted during the study.

### Provisions for post-trial care {30}

This study does not have compensation insurance because it aims to show the non-inferiority of not administering antibiotic prophylaxis. If the enforcement of this study causes a patient to develop a health hazard, it will be treated at the relevant hospital. The costs of the treatment of the health hazard will be borne by the patient’s health insurance.

### Outcomes {12}

The primary outcome is the number of patients that exhibit positive urine cultures (>10^5^ colony-forming units/ml) 3 or 4 days after the renal biopsy, or at which point the patients are diagnosed with pyelonephritis until 3 or 4 days after the renal biopsy. The secondary outcomes of this study include the number of patients who are diagnosed with pyelonephritis within 30 days after the renal biopsy, the number of patients who are diagnosed with a puncture site infection within 30 days after the renal biopsy, the number of patients who are diagnosed with an infection other than pyelonephritis or a puncture site infection within 30 days after the renal biopsy, and the number of patients who experience cefazolin-induced side effects.

Pyelonephritis is defined as a fever of >38 °C and a white blood cell count of >10/HPF according to urinalysis. Urine cultures will be conducted at diagnosis where possible. A puncture site infection is defined as the presence of purulent discharge, spontaneous pain, tenderness, swelling, redness, and/or a feeling of heat at the puncture site. When purulent discharge is present, a culture test will be conducted if possible.

### Participant timeline {13}

Additional file is attached.

### Sample size {14}

The study sample size was calculated in accordance with the 1:1 allocation rule. We anticipated that the urine culture positivity rate would be 1% and the non-inferior margin would be 3%. The urine culture positivity rate was estimated based on our preliminary data. The non-inferior margin was estimated based on a discussion among our group. The purpose of this study is to ensure that the use of antibiotic prophylaxis in renal biopsy patients in Japan is consistent with international standards. Therefore, we think that a slightly large non-inferior margin is acceptable. Assuming an alpha value of 0.05 and a beta value of 0.2 for a two-sided test, 346 cases are required in each group. It is expected that 5% of cases will be inadequate for the purposes of the study, and hence, the number of required cases was set at 364. The software used to determine the sample size was Easy R.

### Recruitment {15}

The recruitment period runs from 7 November 2020 to 31 March 2023. Patients will be recruited at Osaka Red Cross Hospital, Kobe City Medical Center General Hospital, Kyoto Min-Iren Chuo Hospital, National Hospital Organization Kyoto Medical Center, Kitano Hospital, and Kyoto University Hospital. Over 200 percutaneous renal biopsies are carried out at these hospitals annually. The doctors at outpatient clinics refer patients to our hospital. We always cooperate with them.

## Assignment of interventions: allocation

### Sequence generation {16a}

Patients will be randomized to receive either a single dose of intravenous cefazolin (1 g) or no antibiotic prophylaxis (1:1 ratio) using a stratified block allocation, with sex (male or female) being used as a stratification factor. The allocation sequence was generated by a computer using Stata's random-number generator.

### Concealment mechanism {16b}

The results of the allocation process will be concealed from all investigators except for the randomizer.

### Implementation {16c}

After obtaining signed informed consent forms, the investigators will use the assignment list to allocate each patient to one of the study arms. The study group will be revealed at the same time to both the patients and investigators.

## Assignment of interventions: Blinding

### Who will be blinded {17a}

The patients, investigators, and doctors will not be blinded since this is impossible due to the difference between the two groups (cefazolin group versus no prophylaxis group). The statisticians conducting the data analysis will be blinded with regard to the primary and secondary endpoints.

### Procedure for unblinding if needed {17b}

The study will have an open-label design; therefore, there is no unblinding procedure.

## Data collection and management

### Plans for assessment and collection of outcomes {18a}

All clinical data and patient-reported information will be entered into electronic case-record forms. The case-record forms can be obtained by emailing the corresponding author of this manuscript.

### Plans to promote participant retention and complete follow-up {18b}

Patients will be in close contact with the doctors, who will monitor their progression during study visits. If a patient does not come to the hospital as scheduled, we will contact them about their condition via telephone or a sealed letter.

### Data management {19}

The data will be entered into an electronic file on a password-protected computer and will not be accompanied by any identifying information (each patient will be assigned an encrypted ID number). Trained inputters will enter the data, and the participating investigators will check all of the data. The software’s range check system will be used to ensure the quality of the data. The original copies of the instruments will be filed and stored, under lock and key, in a self-storage unit, along with a list of the patients’ names and ID numbers. All measures will be taken to create a backup of the stored data to prevent data loss.

### Confidentiality {27}

The study data will be stored using a study identification code for each participant. The key to the identification code list will only be available to the study team during the study and will be documented and safeguarded by the principal investigator according to study guidelines after the completion of the study. No patient identification details will be reported in publications.

### Plans for collection, laboratory evaluation, and storage of biological specimens for genetic or molecular analysis in this trial/future use {33}

There are no plans to collect, evaluate, or store biological specimens for genetic or molecular analysis in this study.

## Statistical methods

### Statistical methods for primary and secondary outcomes {20a}

The data will be analyzed using the statistical software “R”. Data regarding the primary objective (the number of patients with positive urine cultures) will be presented as categorical variables. In comparisons between the two groups, one-sided *p*-values for non-inferiority will be calculated using the Farrington-Manning test. Continuous variables will be tested for normality and expressed as x±s values for normally distributed variables or as median (interquartile range) values for non-normally distributed variables. Continuous variables will be compared using the independent *t*-test or Mann-Whitney *U* test. Categorical data will be expressed as rates or percentages. Inter-group comparisons will be performed using Fisher’s exact probability test. Two-sided tests will be used, with *p*-values of <0.05 considered to indicate a statistically significant difference.

### Interim analyses {21b}

There are no interim analyses planned.

### Methods for additional analyses (e.g., subgroup analyses) {20b}

A subgroup analysis in which the patients who underwent continuous or intermittent urinary catheterization just before or after the renal biopsy will be compared with those who did not undergo such catheterization will be performed.

### Methods in analysis to handle protocol non-adherence and any statistical methods to handle missing data {20c}

The primary outcome will be assessed using an intention-to-treat analysis. Sensitivity analysis will be performed to deal with any missing data. If a statistical method is needed to account for missing data relating to the primary or secondary outcomes, multiple imputation will be used.

### Plans to give access to the full protocol, participant level-data and statistical code {31c}

Reasonable access to the protocol and other documents can be gained by contacting the corresponding author.

## Oversight and monitoring

### Composition of the coordinating center and trial steering committee {5d}

The trial steering committee (TSC) will be responsible for the supervision of all aspects of the trial, including the completion of the trial to clinical and ethical standards. The TSC will help the study to run smoothly day to day. The members of the TSC include the principal investigator, selected investigators from each site, and a statistician. They meet once a year and have regular discussions.

### Composition of the data monitoring committee, its role, and reporting structure {21a}

The data monitoring committee (DMC) will ensure the safety of the study participants by monitoring the ethical conduct of the study and adverse events and consider any new data (recently published studies) that may affect the validity of continuing with the study. The DMC will also ensure that the study is conducted according to the protocol and that the data are collected appropriately. The DMC includes an independent chairperson and other independent members.

### Adverse event reporting and harms {22}

An adverse event is defined as any untoward medical occurrence in a clinical investigation patient with or without an association with the study.

A serious adverse event is defined as any untoward medical occurrence at any dose that:

- Results in death or
- Is life-threatening or
- Requires inpatient hospitalization or the prolongation of current hospitalization or
- Results in persistent or significant disability/incapacity or
- Is a medically important event

Medical and scientific judgment will be exercised in deciding whether expedited reporting is appropriate in other situations, such as important medical events that may not be immediately life-threatening or result in death or hospitalization, but may jeopardize the patient or may require intervention to prevent one of the other outcomes listed in the definition above. These events will usually be considered serious.

The investigator will evaluate any adverse events experienced by the patients and record them in a case report. If a sign (including laboratory values) or symptom is included within a particular diagnosis, the diagnosis will be recorded rather than individual signs and symptoms, where possible.

For all patients registered in this study, all adverse events that develop will be recorded and monitored until they are treated or until the end of the observation period.

When an adverse event develops, the investigator will give the patient adequate treatment promptly and follow them up until recovery or relief is confirmed.

### Frequency and plans for auditing trial conduct {23}

Once a year, a monitor from the Ethical Scientific Committee will check the presence and completeness of the investigation files, such as informed consent forms, inclusion and exclusion criteria, and the data collection and storage methods.

### Plans for communicating important protocol amendments to relevant parties (e.g. trial participants, ethical committees) {25}

The investigators, ethical review board, and regulators will be notified of any important modifications to the protocol.

### Dissemination plans {31a}

The results of this study will be disclosed completely in an international peer-reviewed journal. Both positive and negative results will be reported. Patients will receive a layman’s summary of the results if they opt in to receive study-level outcomes.

## Discussion

This randomized controlled trial aims to show the non-inferiority of not administering antibiotic prophylaxis for percutaneous renal biopsy. If this study shows that antibiotic prophylaxis is not needed, it would help to ensure patient safety and prevent the development of antibiotic-resistant bacteria.

There have been few studies about infections related to renal biopsy. One study examined the complications associated with 1812 renal biopsies conducted over 37 years [6]. Urinary tract infection followed gross hematuria in 2 patients and was attributed to vigorous irrigation to remove clots from the bladder. Both patients recovered. Another patient developed transient bacteremia with chills, fever, and hypotension following perforation of the colon during a biopsy. The patient recovered with no further difficulty [6]. Some other more previous cases of infection after a renal biopsy have also been reported. These patients had pyelonephritis before the renal biopsy [7, 8].

Catheter-associated urinary tract infections (CAUTI) are the most common type of healthcare-associated infection. There was a marked reduction in risk of bacteriuria after the introduction of sterile, closed urinary drainage systems in the 1960s [9]. However, even when a closed drainage system is used, bacteriuria inevitably occurs over time either via breaks in the sterile system or via the extraluminal route. The daily risk of bacteriuria during catheterization is 3% to 10% and approaches 100% after 30 days [9]. The causative pathogens of CAUTI include *Escherichia coli*, *Candida* spp, and *Enterococcus* spp [9]. These pathogens may be different from the sources of infection for percutaneous renal biopsy-associated infections. Therefore, a subgroup analysis in which the patients who underwent continuous or intermittent urinary catheterization just before or after the renal biopsy will be compared with those who did not undergo such catheterization will be performed.

## Trial status

Recruiting started in November 2020. The current protocol is version 3 of 15 November 2021. It is estimated that patient recruitment will be completed around March 2023.

## Supplementary Information

**Additional file 1.**

## Data Availability

The datasets used and/or analyzed during the current study will be made available from the corresponding author upon reasonable request.

## Abbreviations

CAUTI: Catheter-associated urinary tract infections
HPF: High-power field
